# Identification of novel long non-coding RNAs in clear cell renal cell carcinoma

**DOI:** 10.1186/s13148-015-0047-7

**Published:** 2015-02-08

**Authors:** Jasmine JC Blondeau, Mario Deng, Isabella Syring, Sarah Schrödter, Doris Schmidt, Sven Perner, Stefan C Müller, Jörg Ellinger

**Affiliations:** Department of Urology, University Hospital Bonn, Bonn, Germany; Institute of Pathology, University Hospital Bonn, Bonn, Germany; Department of Prostate Cancer Research, University Hospital Bonn, Bonn, Germany; Center of Integrated Oncology, University Hospital Bonn, Bonn, Germany

## Abstract

**Background:**

Long non-coding RNAs (lncRNA) play an important role in carcinogenesis; knowledge on lncRNA expression in renal cell carcinoma is rudimental. As a basis for biomarker development, we aimed to explore the lncRNA expression profile in clear cell renal cell carcinoma (ccRCC) tissue.

**Results:**

Microarray experiments were performed to determine the expression of 32,183 lncRNA transcripts belonging to 17,512 lncRNAs in 15 corresponding normal and malignant renal tissues. Validation was performed using quantitative real-time PCR in 55 ccRCC and 52 normal renal specimens. Computational analysis was performed to determine lncRNA-microRNA (MiRTarget2) and lncRNA-protein (catRAPID omics) interactions. We identified 1,308 dysregulated transcripts (expression change >2-fold; upregulated: 568, downregulated: 740) in ccRCC tissue. Among these, aberrant expression was validated using PCR: lnc-BMP2-2 (mean expression change: 37-fold), lnc-CPN2-1 (13-fold), lnc-FZD1-2 (9-fold), lnc-ITPR2-3 (15-fold), lnc-SLC30A4-1 (15-fold), and lnc-SPAM1-6 (10-fold) were highly overexpressed in ccRCC, whereas lnc-ACACA-1 (135-fold), lnc-FOXG1-2 (19-fold), lnc-LCP2-2 (2-fold), lnc-RP3-368B9 (19-fold), and lnc-TTC34-3 (314-fold) were downregulated. There was no correlation between lncRNA expression with clinical-pathological parameters. Computational analyses revealed that these lncRNAs are involved in RNA-protein networks related to splicing, binding, transport, localization, and processing of RNA. Small interfering RNA (siRNA)-mediated knockdown of lnc-BMP2-2 and lnc-CPN2-1 did not influence cell proliferation.

**Conclusions:**

We identified many novel lncRNA transcripts dysregulated in ccRCC which may be useful for novel diagnostic biomarkers.

**Electronic supplementary material:**

The online version of this article (doi:10.1186/s13148-015-0047-7) contains supplementary material, which is available to authorized users.

## Background

Renal cell carcinoma (RCC) is one of the most common malignancies; its incidence is varying substantially worldwide: RCC incidence is high in Europe and North America and low in Asia and South America [1]. Today, many small-sized renal tumors are diagnosed; however, imaging modalities do not allow precise differentiation between renal cell carcinoma and non-malignant renal tumors. Performing tumor biopsy and histopathological classification is sometimes challenging, and definitive exclusion of malignancies still requires surgical exploration. As active surveillance protocols for small renal lesions find the way into daily clinical practice, a better estimation of tumor aggressiveness becomes necessary. Thus, identifying novel biomarkers would be helpful for the management of patients with renal tumors.

Nucleic acids are under discussion as potential biomarkers for patients with RCC [2]. Long non-coding RNAs (lncRNA) are a class of RNA molecules arbitrarily defined as being longer than 200 nucleotides and not translated into a protein. Initially, it was thought that lncRNAs represent transcriptional noise, but it is nowadays recognized that lncRNAs may have biological roles. They are regulating imprinting, dosage compensation, cell cycle, pluripotency, retrotransposon silencing, and meiotic entry, for example [3]. However, knowledge on lncRNA expression and their function is still in its infancy, but lncRNA expression seems to be highly tissue and tumor specific [4]. So far, few are known on lncRNAs expression in RCC [5-8]. We therefore performed microarray experiments to study the expression of 32,183 lncRNA transcripts in a cohort of patients with clear cell RCC (ccRCC) to determine a comprehensive lncRNA profile.

## Results

### Microarray: screening for aberrantly expressed lncRNAs

**Figure 1:**
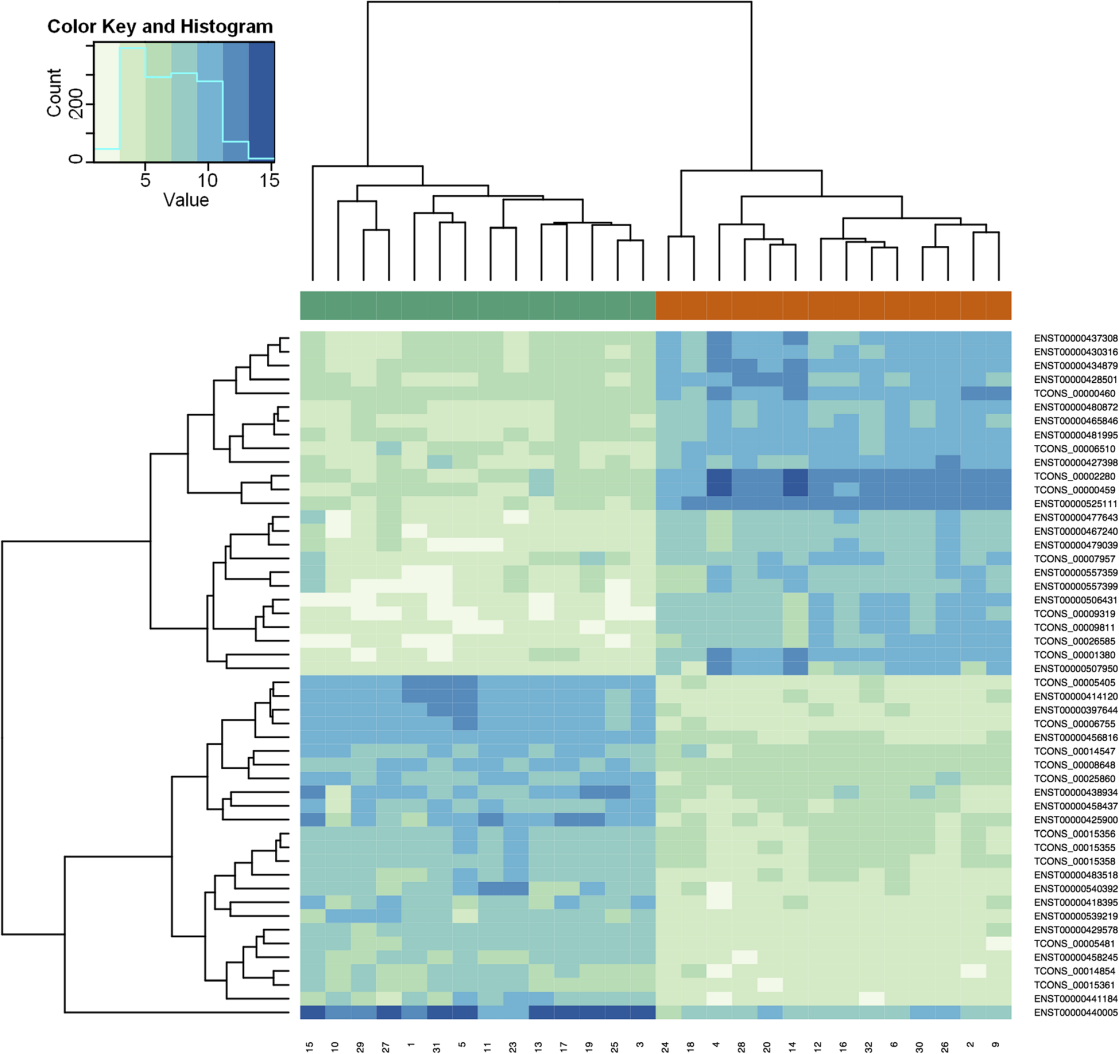
**lncRNA expression profiling.** The expression of 32,183 lncRNA transcripts was determined in 15 corresponding clear cell renal carcinoma (ccRCC) and normal renal tissue samples. The expression profile of 25 different lncRNA transcript variants allows accurate discrimination of normal and malignant renal tissue as shown in the heatmap. All ccRCC tissues (green bar above the heatmap) and normal renal tissues (red bar) are located in a separate cluster.

A gene expression microarray was used to identify dysregulated lncRNAs in 15 corresponding tumor and normal renal tissue samples as a discovery cohort. Among the 32,183 analyzed lncRNA transcripts, we observed differential expression (fold change >2) in 1,308 transcripts: 568 lncRNA transcripts were upregulated and 740 were downregulated in ccRCC samples. The 20 most differentially expressed lncRNAs in ccRCC and normal renal tissue are listed in Table 1. A hierarchical cluster analysis based on centered Pearson correlation coefficient was performed to determine different expression profiles in ccRCC and normal tissue. As shown in Figure 1, the lncRNA expression profile allowed distinguishing cancerous and normal tissue samples highly accurately.

**Table 1:** **List of 20 differentially expressed lncRNAs in renal cell carcinoma identified using a microarray screening in each 15 normal and malignant renal tissues**

**Downregulated in cancer**		**Upregulated in cancer**	
**lncRNA**	**Log2 fold change**	**lncRNA**	**Log2 fold change**
lnc-TTC34-3	−6.585	lnc-CPN2-1	5.966
lnc-ACACA-1	−6.486	lnc-BMP2-2	4.787
lnc-LCP2-2	−5.771	lnc-DGCR6-2	4.659
lnc-AC068473.1-2	−5.654	lnc-ITPR2-3	4.379
lnc-MED10-7	−5.145	lnc-SLC30A4-1	4.150
lnc-TMEM18-8	−4.880	lnc-NR2E1-1	4.131
lnc-ATP1A1OS-2	−4.770	lnc-FZD1-2	4.019
lnc-FOXG1-2	−4.485	lnc-DGCR6-2	3.920
lnc-TRAT1-2	−4.484	lnc-PADI1-1	3.904
lnc-RP3-368B9.1.1-1	−4.453	lnc-SPAM1-6	3.516
lnc-IFRD2-1	−4.440	lnc-PRRC2C-1	3.508
lnc-TBCCD1-1	−4.369	lnc-MYC-2	3.483
lnc-C15orf2-2	−3.981	lnc-CDRT15L2-1	3.479
lnc-ATP8A1-1	−3.960	lnc-SCRG1-1	3.275
lnc-LY86-3	−3.876	lnc-TMEM30B-5	3.260
lnc-SCN2A-2	−3.787	lnc-RCBTB2-1	3.205
lnc-ST6GALNAC3-1	−3.786	lnc-AHR-1	3.034
lnc-MIS18BP1-1	−3.557	lnc-DLX2-4	2.903
lnc-RASL11B-2	−3.532	lnc-HPS3-2	2.720
lnc-RACGAP1-2	−3.528	lnc-RCBTB2-3	2,709

### Real-time PCR: validation of expression profiling

**Figure 2:**
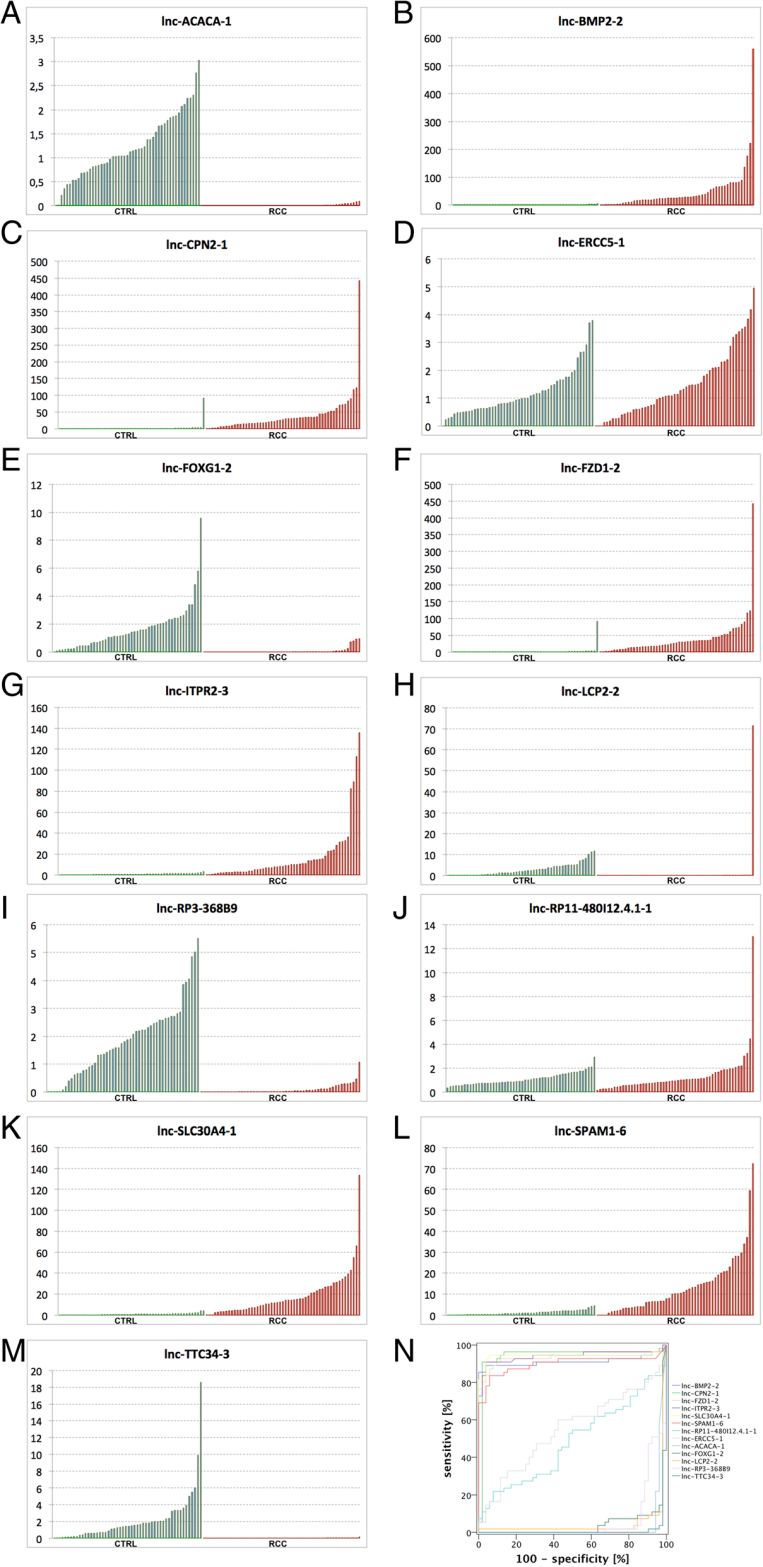
**Validation of lncRNA dysregulation.** The expression of 13 target lncRNAs in renal cell carcinoma (red) and normal renal (green) tissue was validated using quantitative real-time PCR; the expression levels were normalized using ACTB and PPIA as reference genes. We confirmed significant (all *p* < 0.001) overexpression of lnc-BMP2-2 **(B)**, lnc-CPN2-1 **(C)**, lnc-FZD1-2 **(F)**, lnc-ITPR2-3 **(G)**, lnc-SLC30A6-1 **(K)**, and lnc-SPAM1-6 **(L)** in renal cell carcinoma, whereas lnc-ACACA-1 **(A)**, lnc-FOXG1-2 **(E)**, lnc-LCP2-2 **(H)**, lnc-RP3-368B9 **(I)**, and lnc-TTC34-3 **(M)** were significantly downregulated (all *p* < 0.001); lnc-ERCC5-1 (**D**, *p* = 0.401) and lnc-RP11-480I12.4.1-1 (**J**, *p* = 0.731) were not different in malignant and normal renal tissue. Receiver operator characteristic **(N)** analyses demonstrate excellent discrimination of the lncRNAs between both cohorts (area under the curve 0.90–0.94).

In order to confirm aberrant lncRNA expression, we determined the expression of 13 lncRNAs in an independent validation cohort of ccRCC (*n* = 55) and normal (*n* = 52) renal tissue samples. The selection of representative lncRNAs for validation was based on the degree of dysregulation in the microarray (six upregulated and five downregulated lncRNA; in addition two non-regulated lncNRA). As expected, lnc-BMP2-2 (mean: 37-fold), lnc-CPN2-1 (13-fold), lnc-FZD1-2 (9-fold), lnc-ITPR2-3 (15-fold), lnc-SLC30A4-1 (15-fold), and lnc-SPAM1-6 (10-fold; all *p* < 0.001) were highly overexpressed in RCC, whereas lnc-ACACA-1 (135-fold), lnc-FOXG1-2 (19-fold), lnc-LCP2-2 (2-fold), lnc-RP3-368B9 (19-fold), and lnc-TTC34-3 (314-fold) were downregulated (all *p* < 0.001). Both, lnc-ERCC5-1 (*p* = 0.401) and lnc-RP11-480I12.4.1-1 (*p* = 0.731) were—as observed in the microarray studies—not dysregulated in tumor samples. The lncRNA expression levels allowed highly sensitive and specific discrimination between RCC and control subjects as determined by using ROC analyses: the area under the curve was >0.9 for all dysregulated samples, especially lnc-CPN2-1 overexpression allowed molecular identification of RCC tissue (AUC 0.942, 95% confidence interval 0.884–1.000). See Figure 2 and Table 2.

**Table 2:** **Receiver operator characteristic analyses for discrimination of normal and malignant renal tissue based on lncRNA expression**

**lncRNA**	**Area under the curve**	**95% confidence interval**
lnc-FZD1-2 upregulation	0.931	0.871–0.991
lnc-SLC30A4-1 upregulation	0.942	0.883–1.000
lnc-BMP2-2 upregulation	0.912	0.843–0.981
lnc-SPAM1-6 upregulation	0.900	0.830–0.969
lnc-ITPR2-3 upregulation	0.941	0.887–0.994
lnc-CPN2-1 upregulation	0.942	0.884–1.000
lnc-TTC34-3 downregulation	0.990	0.973–1.000
lnc-ACACA-1 downregulation	0.966	0.923–1.000
lnc-LCP2-2 downregulation	0.955	0.906–1.000
lnc-FOXG1-2 downregulation	0.954	0.911–0.997
lnc-RP3-368B9.1.1-1 downregulation	0.938	0.892–0.984

We also analyzed whether lncRNA expression levels were correlated with poor prognostic parameters; the Bonferroni method was applied to correct for multiple hypothesis testing (adjusted significance value *p* < 0.0083). None of the lncRNAs was significantly correlated with staging nor grading. However, there was a tendency towards lower lnc-ERCC5-1 (*p* = 0.034) and RP3-368B9 (*p* = 0.016) levels in Fuhrman grade 3/4 tumors, lower lnc-ERCC5-1 (*p* = 0.026) expression in vascular invasive tumors, and advanced AJCC stage in samples with low lnc-RP3-368B9 (*p* = 0.011). lncRNAs were not associated with progression-free survival, overall survival or cancer-specific survival (all p > 0.05, data not shown), as determined using Cox regression analysis.

### siRNA-mediated lncRNA knockdown

**Figure 3:**
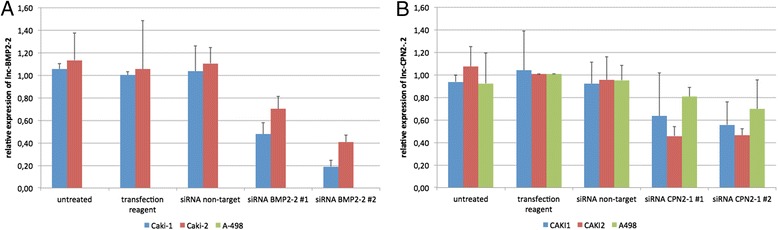
**lncRNA expression after siRNA-mediated lncRNA knockdown.** Expression of lnc-BMP2-2 **(A)** and lnc-CPN2-1 **(B)** was manipulated using specific siRNA in Caki-1, Caki-2, and A-498 renal cell carcinoma cell lines. A-498 did not express lnc-BMP2-2. lnc-BMP2-2 expression was significantly (*p* < 0.05) decreased in siRNA-treated Caki-1 and Caki-2 cells; lnc-CPN2-1 was also decreased in Caki-2 cells.

**Figure 4:**
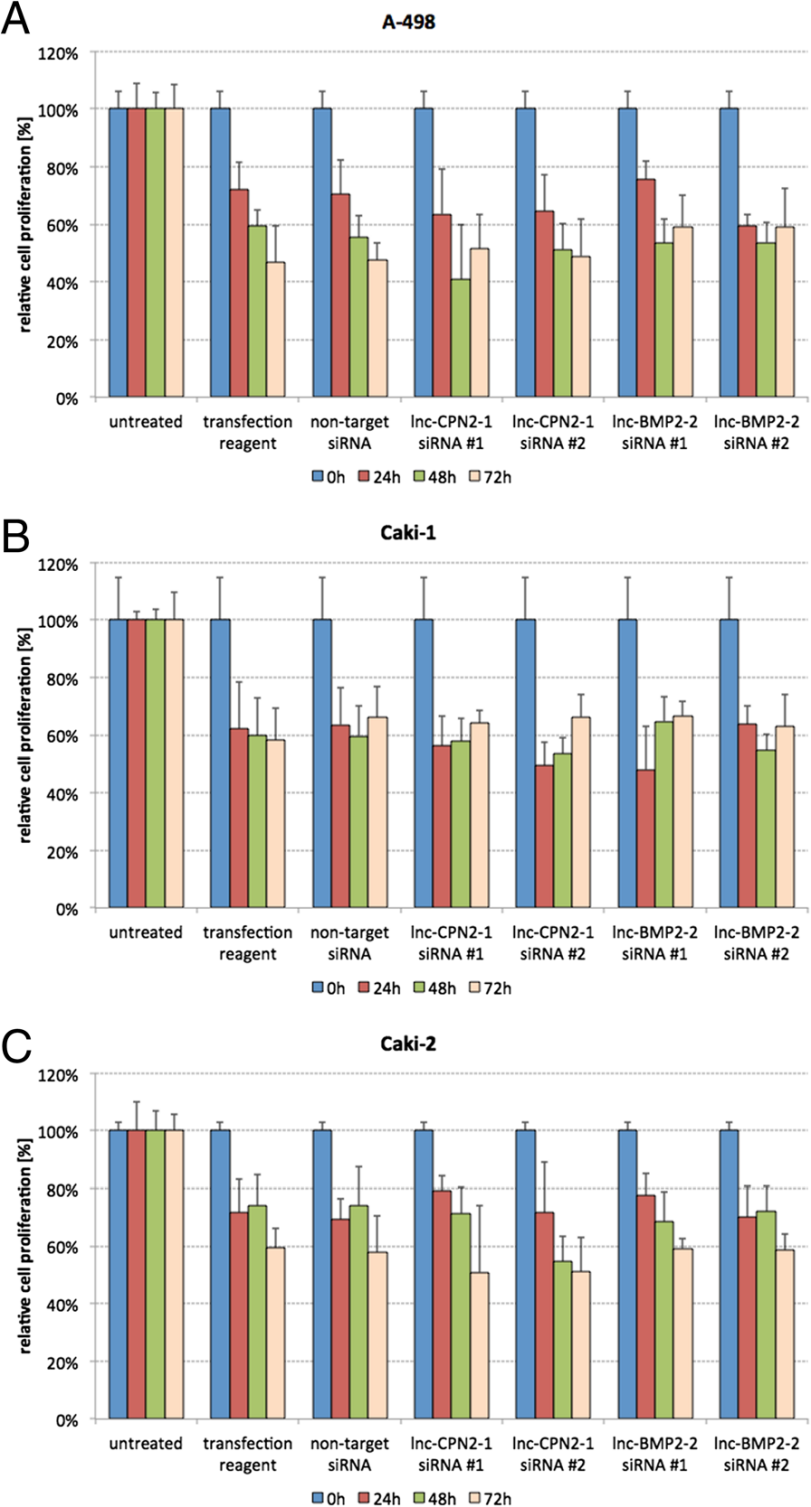
**Cell proliferation after siRNA-mediated lncRNA knockdown.** The EZ4U test was applied for cell proliferation testing; proliferative activity was not changed in any renal cell carcinoma cell line (**A**, A-498; **B**, Caki-1; **C**, Caki-2).

We next treated Caki-1, Caki-2, and A-498 RCC cell lines with small interfering RNA (siRNA) targeting lnc-BMP2-2 and lnc-CPN2-1; see Figure 3. lnc-BMP2-2 expression was significantly (*p* < 0.05) decreased in siRNA-treated Caki-1 and Caki-2 (only siRNA #2) cells; it was basically unexpressed in A-498 cells. lnc-CPN2-1 was also decreased in Caki-2 cells (*p* < 0.05). Glyceraldehyde 3-phosphate dehydrogenase (GAPDH) expression was significantly reduced in all cell lines (not shown); the negative controls did not change gene expression. However, the EZ4U test did not show any changes in cellular proliferative activity after siRNA-mediated lncRNA knockdown. See Figure 4.

### Computational prediction of microRNA targeting and protein interaction

The MiRTarget2 algorithm [9] was used to predict microRNA seeds within the validated lncRNA transcripts. Most transcripts do not have any (lnc-BMP2-2; lnc-FZD1-2; lnc-ITPR2-3:1; lnc-SPAM1-6; lnc-TTC34-4) or only few (lnc-ACACA-1: miR-4652-3p, miR-5001-3p, miR-4753-3p; lnc-CPN2-1: miR-149-3p; lnc-LCP2-2: miR-3191-5p; lnc-SLC30A4-1: miR-4772-5p) predicted targeting microRNAs with—so far—unknown functions. lnc-FOXG1-2 has multiple predicted target microRNAs (miR-3662, miR-3120-5p, miR-519c-3p, miR-519a-3p, miR-548ag, miR-4282, miR-519b-3p, miR-458 m, miR-3163, miR-4658, miR-676-5p, miR-3973). It was reported that lncRNAs can act as a microRNA sponge by binding specific microRNAs and thereby by interfering with their role as regulator of gene expression [10]. However, none of the predicted microRNA targets of FOXG1-2 was correlated to FOXG1-2 levels in a cohort of each ten normal and malignant renal tissue samples (data not shown; miR-519a, miR-519b, miR-519c, miR-548, miR-3120, miR-4284, miR-4658: all *p* > 0.1). MicroRNA expression was similar in RCC and normal renal tissue (all *p* > 0.5).

Furthermore, we determined lncRNA-protein interactions using the catRAPID omics algorithm [11]. Numerous lncRNA-protein interactions (*n* = 91) are predicted for all transcripts (see Additional file 1: Table S2); the cellular functions of proteins with a ranking score >2.5 were next explored using GeneMANIA [12]. The interacting networks are mainly related to splicing, binding, transport, localization, and processing of RNA but also transcription and translation (see Table 3; Additional file 2: Table S3).

**Table 3:** **Computationally predicted functions by lncRNA-protein interactions**

**Predicted function**	**lnc-ACACA-1**	**lnc-BMP2-2**	**lnc-CPN2-1**	**lnc-FOXG1-2**	**lnc-FZD1-2**	**lnc-ITPR2-3**	**lnc-LCP2-2**	**lnc-RP3-368B9.1.1-1**	**lnc-SLC30A4-1**	**lnc-SPAM1-6**	**lnc-TTC34-3**
mRNA processing	X	X	X	X	X	X	X	X	X	X	X
mRNA/RNA splicing	X	X	X	X	X	X	X	X	X	X	X
mRNA binding		X	X	X	X	X	X		X	X	X
RNA localization	X	X				X	X	X	X	X	X
Ribonucleoprotein complex		X			X	X	X	X	X		X
RNA transport	X					X	X	X	X	X	X
Transcription	X					X	X	X	X	X	X
Posttranscriptional regulation		X	X	X	X	X		X			
mRNA/RNA stabilization		X	X	X	X	X					
DNA binding						X				X	X
Translation					X		X		X		
Protein localization									X		
rRNA processing									X		
Viral genome expression									X		

## Discussion

lncRNA are important regulators of gene expression during genetic information processing in living cells and interact with major cellular pathways (e.g. proliferation, differentiation, apoptosis). Thereby, they are involved in carcinogenesis of many human malignancies [13], but so far, knowledge about lncRNA expression in RCC is limited [5-8]. We determined the expression of 32,183 lncRNA transcripts belonging to 17,512 lncRNAs in 15 corresponding normal and malignant renal tissues, thereby providing the most comprehensive analysis of lncRNA expression in RCC up to now. We observed differential expression of 1,308 lncRNA transcripts (defined as expression differences >2-fold) corresponding to 4.1% of the studied transcripts. The expression of lncRNAs was successfully validated for upregulated (lnc-BMP2-2, lnc-CPN2-1, lnc-FZD1-2, lnc-ITPR2-3, lnc-SLC30A4-1, lnc-SPAM1-6), downregulated (lnc-ACACA-1, lnc-FOXG1-2, lnc-LCP2-2, lnc-RP3-368B9, lnc-TTC34-3), and unregulated (lnc-ERCC5-1, lnc-RP11-480I12.4.1-1) transcripts using qPCR in a cohort of 55 ccRCC and 52 normal renal specimen, thus the specificity of the microarray results is confirmed. Two recent studies investigated the lncRNA expression profile of RCC tissues using microarray technologies, but samples sizes were small (*n* = 4 [5]; *n* = 6 [6]; *n* = 11 [7]), thereby limiting powerful statistical analysis; furthermore, the analyses were limited to a set of 984 lncRNAs in the Fachel study [7]. During later courses of our study, Malouf et al. [8] re-analyzed the data obtained in The Cancer Genome Atlas project and identified 1,934 lncRNA expressed in RCC. The catalog of the differentially expressed lncRNAs varied in the profiling studies, but notably, all studies demonstrated a tendency towards an increased ratio of down- to upregulated lncRNAs [6,7]. Earlier studies linked aberrant expression of individual lncRNAs to RCC in small-scaled studies (*n* ≤ 12; i.e. GAS5, also termed SERPINC1 [8,14]; aHIF [15]; MALAT1 [8]); we could not confirm GAS5 downregulation in our microarray study; aHIF and MALAT1 was not deregulated.

lncRNAs could serve as diagnostic and prognostic biomarkers: The distinct overexpression of lncRNAs in RCC could be used for the development of a non-invasive diagnostic biomarker. We demonstrated distinct (>10-fold) expression level differences for a number of lncRNAs including lnc-ACACA-1, lnc-BMP2-2, lnc-CPN2-1, lnc-FOXG1-2, lnc-FZD1-2, lnc-ITPR2-3, lnc-RP3-368B9, lnc-SLC30A4-1, lnc-SPAM1-6, and lnc-TTC34-3; if expression differences are validated by independent researchers, these lncRNAs could represent diagnostic biomarkers. lncRNAs are detectable in bodily fluids and may thereby serve for cancer diagnosis: The PROGENSA® PCA3 Assay utilizes the detection of the lncRNA PCA3 in urine as diagnostic biomarker for prostate cancer [16]. Furthermore, plasma levels of HULC were increased in hepatocellular carcinoma [17], H19 in gastric cancer [18], and MALAT-1 in prostate cancer [19] patients.

In addition, lncRNAs may be of prognostic relevance: Fachel et al. [7] reported a 26-gene lncRNA expression profile, but not a single lncRNA, associated with the survival of ccRCC patients. However, the small sample size (*n* = 16) and the lack of a validation cohort limit the informative value of the study [7]. Malouf et al. [8] identified four lncRNA expression clusters among the study cohort, and the survival was significantly reduced in one subgroup (C2). We did not observe a correlation of lncRNAs (used in the validation study) with any clinicopathological parameter nor survival data. Reasons for the failure to identify prognostic relevant lncRNAs include: (*i*) a microarray study not powered for the identification of expression differences in localized (*n* = 5) and advanced RCC (*n* = 10), (*ii*) the selection of lncRNAs for validation based on the differential expression of tumor and normal tissue samples, and (*iii*) the high number of patients with localized ccRCC (56% AJCC stage 1 or 2) in the validation cohort. We expect that future studies designed to identify prognostic relevant lncRNAs in RCC patients will be able to present some candidates, because altered expression of specific lncRNAs was predictive of cancer-specific survival in former studies on other human malignancies; i.e. upregulation of HOTAIR in breast cancer [20] and SChLAP1 in prostate cancer patients [21] was associated with a poor prognosis.

As interest in lncRNA research has increased a short time ago, the function of lncRNAs remains largely unknown, but involvement in many fundamental cellular processes is assumed [13]. The short half-life time (<2 h) of nuclear lncRNA predisposes for regulative functions [22,23]. We performed *in silico* analyses to point out putative functions of dysregulated lncRNAs: (*i*) lncRNA may act as miRNA sponge (e.g. miR-372 expression is downregulated by the lncRNA HULC [24]). However, the expression levels of several FOXG1-2 target microRNAs were not correlated to the level of FOXG1-2, thus we cannot confirm such a role for the investigated lncRNAs. (*ii*) Furthermore, lncRNA may function via lncRNA-protein interactions [25]. Using catRAPID omcis [11], we identified putative 91 lncRNA-protein interactions for the dysregulated lncRNAs (lnc-ACACA-1, lnc-BMP2-2, lnc-CPN2-1, lnc-FOXG1-2, lnc-FZD1-2, lnc-ITPR2-3, lnc-LCP2-2, lnc-RP3-368B9, lnc-SLC30A4-1, lnc-SPAM1-6, lnc-TTC34-3). Most of these proteins are functioning in splicing, binding, transport, localization, transcription, translation, and processing of RNA. Providing experimental evidence beyond *in silico* predictions is necessary in future studies.

Somewhat surprisingly, neither siRNA-mediated knockdown of lncRNA-CPN2-2 nor lnc-BMP2-2 resulted in a significant reduction of cell proliferation. However, the lncRNA knockdown was of moderate success (relative expression change in siRNA-treated cell lines 2- to 5-fold, despite of the extensive optimization of siRNA treatment in preliminary experiments), and thus the toxicity of the transfection reagent may inhibit determining the functional relevance of both lncRNAs. Many lncRNAs regulate nuclear events and must therefore be localized in the nucleus [26]. As is known, the knockdown of nuclear RNAs is often difficult [27], and therefore our efforts to determine the functional relevance of lncRNAs failed.

## Conclusions

In summary, our study reveals that dysregulation of approximately 4% of the lncRNA transcripts occurs in ccRCC, and altered lncRNA expression may modulate fundamental cellular processes. lncRNA profiles allow to accurately distinguish ccRCC and normal renal tissue. Thus, lncRNAs may be used as non-invasive biomarker for RCC patients.

## Methods

### Patients

Biomaterials including fresh-frozen tissues are prospectively collected in the Biobank at the CIO Köln Bonn at the Universitätsklinikum Bonn according to standard operating procedures. Tissue from patients undergoing radical nephrectomy or nephron-sparing surgery was snap frozen, and samples from tumor and normal renal tissue were stored at liquid nitrogen. Hematoxylin- and eosin-stained sections were performed to confirm the histology of the samples. The final histological diagnosis was made on the formalin-fixed paraffin-embedded tissue samples. All cases used in this study were reviewed by an experienced uropathologist; the TNM classification (7th edition from 2009) was applied. We used a discovery cohort with 15 patients (ccRCC and adjacent normal renal tissue) for screening 32,183 lncRNA transcripts; the independent validation cohort consisted of 55 ccRCC and 52 normal renal tissue samples. The detailed clinical-pathological parameters are reported in Table 4. All patients gave written informed consent prior collection of biomaterials. The study was approved by the Ethikkommission at the Universitätsklinikum Bonn (number: 280/12).

**Table 4:** **Summary of clinicopathological parameters of the study cohort**

	**Screening cohort**	**Validation cohort**	
	***n*** **= 15 (%)**	**Cancer** ***n*** **= 55 (%)**	**Normal** ***n*** **= 52 (%)**
*Sex*			
Male	10 (66.6)	37 (67.3)	36 (69.2)
Female	5 (33.3)	18 (33.7)	16 (30.8)
*Age*			
Mean	61.2	62.9	62.1
Min-max	43–86	36–86	36–86
*Pathological stage*			
pT1	4 (26.7)	29 (52.7)	n.a.
pT2	2 (13.3)	7 (12.7)	n.a.
pT3	9 (60.0)	18 (32.7)	n.a.
pT4	0 (0)	1 (1.8)	n.a.
Vascular invasion	7 (46.7)	16 (29.1)	n.a.
Lymph node metastasis	0 (0)	2 (3.6)	n.a.
Distant metastasis	1 (6.7)	7 (12.7)	n.a.
*AJCC staging group*			
I	4 (26.7)	26 (47.3)	n.a.
II	1 (6.7)	5 (9.1)	n.a.
III	9 (60.0)	16 (29.1)	n.a.
IV	1 (6.7)	8 (14.5)	n.a.
*Fuhrman grading*			
Grade 1	2 (13.3)	4 (7.3)	n.a.
Grade 2	11 (73.3)	41 (74.5)	n.a.
Grade 3	2 (13.3)	9 16.3)	n.a.
Grade 4	0 (0)	1 (1.8)	n.a.

*n.a.* not applicable.

### Cell culture and siRNA experiments

The cell lines Caki-1, Caki-2, and A-498 were obtained from the DSMZ (Braunschweig, Germany). All cell lines were maintained at 37°C, 5% CO_2_ in RPMI 1640 culture medium, supplemented with 10% heat-inactivated fetal calf serum and 2 mM of glutamine (PAA, Pasching, Austria). The knockdown of lnc-BMP2-2 (#1: sense GCG-UUU-UAA-UGU-CCA-CCA-Att, antisense UUG-GUG-GAC-AUU-AAA-ACG-Caa; #2: sense GAA-AGA-GAC-UGA-AUA-AUU-Att, antisense UAA-UUA-UUC-AGU-CUC-UUU-Ctc) and lnc-CPN2-1 (#1: sense CAC-UCA-UCU-UUA-AAU-UAG-Att, antisense UCU-AAU-UUA-AAG-AUG-AGU-Gat; #2: sense CAA-UGA-AAC-AGA-ACA-GAU-Att, antisense UAU-CUG-UUC-UGU-UUC-AUU-Gtt) was performed with individually designed Ambion Silencer Select siRNAs (Life Technologies, Foster City, CA, USA); two different siRNAs were designed for each target. In addition, negative controls (transfection reagent without siRNA; Silencer Select Negative Control siRNA #1) and a positive control (Silencer GAPDH Positive Control siRNA) were used. The cell lines were seeded at 2.5 × 10^5^ cells/well (6-well plate), and a forward transfection using the Screenfect Transfection Kit (Genaxxon, Ulm, Germany) with a siRNA concentration of 20 pmol was performed according to the manufacturer’s recommendations. The transfection complex was replaced after 24 h with RPMI medium and incubated for up to 72 h. siRNA experiments were confirmed in three independent experiments.

### Cell proliferation assay

The cell lines were seeded at a concentration of 1.5 × 10^4^ cells per well into a 96-well plate. After forward transfection (siRNA concentration 1.2 pmol), the cells were cultured up to 72 h to determine the proliferative activity after siRNA-mediated knockdown of lnc-BMP2-2 and lnc-CPN2-1. The EZ4U assay (Biomedica, Vienna, Austria) was used to determine cell viability using a 340 ATTC Spectra Thermo SLT photometer (Crailsheim, Germany) according to the manufacturer’s protocol.

### RNA isolation

Fresh-frozen tissue samples (approximately 50 mg) were cut on liquid nitrogen and homogenized with Yttrium stabilized Zirkonoxid-Beads (Silibeads, Sigmund-Lindner, Warmensteinach, Germany) using a Precellys®24 tissue homogenizer (Peglab, Erlangen, Germany). Total RNA was then isolated using the mirVana miRNA Isolation Kit (Ambion, Foster City, CA, USA) according to the manufacturer’s recommendation. To remove residual DNA fragments, we treated the isolate twice with DNase (DNA-free Kit, Ambion). RNA quantity was determined with a NanoDrop 2000 spectrophotometer (Thermo Scientific, Wilmington, DE, USA). RNA integrity was measured in samples used for microarray experiments using the Bioanalyzer 2100 with a RNA 6000 Nano Kit (Agilent Technologies, Santa Clara, CA, USA); only samples with a RIN >6 were used. RNA degradation was excluded by agarose gel electrophoresis in samples used for PCR.

### Microarray

The microarray experiments were performed by Biogazelle (Zwijnaarde, Belgium) as a contract service. RNA isolates from 15 corresponding normal renal and ccRCC tissues (100 ng total RNA) were sent on dry ice to Biogazelle. A custom microarray (Agilent SurePrint G3 technology) based on LNCipedia 2.1 [10] was used to study 32,183 lncRNA transcripts belonging to 17,512 lncRNAs. Background substracted and normalized (log2-based) expression data were provided from Biogazelle for further data analysis. Expression data of lncRNAs for ccRCC and normal tissues was groupwise normalized deducting group mean value. Pairwise fold change, for each gene, was calculated by dividing ccRCC group mean of RNA expression level by normal group mean of expression levels. Pearson’s correlation coefficient was used for correlation analyses (R-Base Package v3.0.2). A heatmap was created using gplots v2.12.1 and RColorBrewer v1.0-5.

### Real-time PCR

Quantitative real-time PCR was performed to validate the microarray experiments using an independent cohort of 55 ccRCC and 52 normal renal tissue samples and to confirm the success of siRNA-mediated lncRNA knockdown. Complementary DNA (cDNA) was synthesized with 1 μg RNA using the PrimeScript RT Reagent Kit with gDNA Eraser. Real-time PCR was performed with 5 ng cDNA template using the 1× SYBR Premix Ex Taq II with ROX Plus and 10 pmol/μl PCR primers (see Additional file 3: Table S1 for primer sequences); all reagents were from Takara Bio, Saint-Germain-en-Laye, France. PCR was performed using an ABIPrism 7900 HT Fast Real-Time PCR System (Applied Biosystems, Foster City, CA, USA). Data analysis was performed using Qbase + (Biogazelle) with PPIA and ACTB as reference genes in the 2-∆∆CT algorithm. SPSS Statistics v21 (IBM, Ehningen, Germany) was used for statistical analyses (Mann–Whitney-*U* test, Cox regression analysis).

### MicroRNA quantification

Quantitative real-time PCR was used to determine the tissue levels of several FOXG1-2 microRNA targets in each ten normal and malignant renal tissues using the Qiagen miScript SYBR Green PCR technology (Hilden, Germany): 1,000 ng total RNA was reverse transcribed and real-time PCR was performed with 5 ng cDNA template. Pre-designed miScript Primer Assays for miR-519a, miR-519b, miR-519c, miR-548, miR-3120, miR-4284, miR-4658, and SNORD43 were used. PCR was performed using the ABIPrism 7900 HT Fast Real-Time PCR System. Data analysis was performed using Qbase + with SNORD43 as reference genes in the 2-∆∆CT algorithm. IBM SPSS Statistics v21 was used for statistical analyses (Pearson correlation test).

### *In silico* analyses

The MiRTarget2 algorithm [9] was used to predict microRNA seeds within the validated lncRNA transcripts. Potential lncRNA-protein interactions were determined using the catRAPID omics algorithm [11]. The cellular functions of proteins were retrieved with GeneMANIA [12]. Standard settings were used in all software packages.

## Availability of supporting data

The microarray data sets supporting the results of this article are available in the Gene Expression Omnibus repository (data entry: GSE61763).

## Additional files

Additional file 1: Table S2. Summary of predicted lncRNA-protein interactions (catRAPID omics algorithm). Additional file 2: Table S3. Summary of lncRNA interacting networks for cellular functions (GeneMANIA algorithm). Additional file 3: Table S1. List of PCR primers.
